# The Effect of the Grain for Green Program on Ecosystem Health in the Upper Reaches of the Yangtze River Basin: A Case Study of Eastern Sichuan, China

**DOI:** 10.3390/ijerph16122112

**Published:** 2019-06-14

**Authors:** Rui Han, Luo Guo, NuanYin Xu, Dan Wang

**Affiliations:** 1 College of the Life and Environmental Science, Minzu University of China, Beijing 100081, China; 18301234@muc.edu.cn; 2 School of Urban Planning and Design, Peking University, Shenzhen 518055, China; xunuanyin@pku.edu.cn; 3 International Doctoral Innovation Centre, Research Group of Natural Resources and Environment, Department of Chemical and Environmental Engineering, University of Nottingham Ningbo China, Ningbo 315100, China; Dan.Wang@nottingham.edu.cn

**Keywords:** ecosystem health, the Grain for Green Program, the LULC change direction model, Pressure–State–Response model, Eastern Sichuan Region

## Abstract

The Eastern Sichuan Region (ESR) is one of the key pilot regions for Grain for Green Program (GGP) implementation in the upper reaches of the Yangtze River basin in China. Therefore, monitoring the effect of the GGP on the ecosystem in the ESR is important. In this study, the Mann–Kendall Trend Test Model was used to ascertain the changes in vegetation coverage. The transfer matrix was used to explore the changes in Land Use/Land Cover (LULC). LULC change direction model (LCDM) was used to preliminarily assess the impact of LULC changes on the ecosystem. The Pressure–State–Response model (PSR), reflecting the human pressure and the ecosystem state, was applied to analyze the spatial–temporal characteristics of the ecosystem health index (EHI). The time span of this study was from 1990 to 2015. The results show that the vegetation coverage changed significantly (*p* < 0.05), and ecosystem function developed towards positive because of the increase in the coverage of forestland and water land and decrease in the coverage of farmland. The spatial distribution of the EHI was influenced by the pattern of land use. The eastern region, associated with a large area of forestland and grassland, has a low population density and a low degree of land use exploitation, resulting in a high EHI value. The situation was completely opposite in the western region. Regarding the temporal scale, in spite of the decreasing pressure indicator, most counties had experienced an increase in the EHI. There was a clear correlation between the increased EHI values and the restored areas at the third stage (2000–2005) (*p* < 0.05, r^2^ = 0.164), but this correlation disappeared at the latter stage (2005–2015) (*p* > 0.05). The changes showed significant variations in time and area because of differences in the process and the intensity of the implication of the GGP.

## 1. Introduction

In recent decades, the poor management of ecosystems has caused many serious ecological problems throughout the world [1,2]. In China, the world’s largest developing country, protecting the ecological environment and maintaining ecosystem health has always been a governmental concern [3]. The Grain for Green Program (GGP) was launched by the Chinese government in 1999 and implemented in 25 provinces in 2002 to mitigate the effect of soil erosion and restore ecosystems by planting trees on former steep areas of cropland or uncultivated barren land [4,5,6]. From 1999 to 2014, 29.8 million hectares of steep sloping cropland and barren land was afforested, and a further 5.33 million hectares of sloping cropland is planned to be afforested by 2020 [7,8]. The Yangtze River is a vital lifeline in China. The ecosystem condition of its upper reaches critically influences the sustainable development of the whole basin [9]. Therefore, the upper reaches of the Yangtze River are one of the key areas for the GGP in China [10]. The soil erosion has been very serious in the upper reaches of the Yangtze River in recent years and has directly caused flooding in the lower reaches [11]. The GGP, which converts cultivated land in the steep hills back into forestland and bans deforestation in the upper reaches of the Yangtze River, has a long-standing ecological significance.

In previous research, the effects of the GGP on its areas of implementation have been assessed with a main focus on the fundamental ecosystem structure, such as the change in vegetation coverage [12,13] and the landscape pattern [14]. Some studies also assessed the effect of ecological restoration on ecological service, but limited to the ecological services, such as water and soil conservation [15], enhancement of soil fertility and carbon sequestration [16], and climate regulation [17]. The challenge of assessing the effect of the GGP is quantitatively assessing ecosystem health under the background of government programs against human pressure and ecosystem state factors. Therefore, an assessment system that can be widely applied is required. Methodologies and indices of ecosystem health from an ecological point of view offer alternative options for evaluating the effects of the GGP, as well as the interaction between human activities and ecosystems corresponding to the implementation process of the GGP [18]. Thus, exploring the effects of the GGP on the change in ecosystem health is of great significance to the upper reaches of the Yangtze River Basin.

The objectives of the GGP are to protect the existing forests and to increase vegetation coverage by means of afforestation. Analyzing whether the GGP has changed the trend in vegetation coverage is the basis of assessing its impact on ecosystem health [19]. The term “ecosystem health” was first defined by Rapport, who defined a healthy ecosystem as having the ability to maintain an organizational structure, and to recover after disturbances in self-regulating processes [20,21]. A variety of methods have been applied to quantify the quality and value of ecosystem health. The following evaluation methods are all widely used to characterize ecosystem health: landscape development intensity (LDI) [22], hydrogeomorphic approaches (HGM) [23], indices of biological integrity (IBI) [24], landscape pattern indices [25], and Pressure–State–Response (PSR) modeling methods [18]. The PSR model, which was fully developed by the Organization for Economic Cooperation and Development, based on the interaction between human and environmental factors [26], provides a systematic mechanism to monitor the status of the sustainable development of ecosystems. The PSR model clearly describes the overall ecosystem health and the relationship between the cause and effect, which have always been emphasized [27,28].

The Eastern Sichuan Region (ESR) is distributed at the end of the upper reaches of the Yangtze River basin. The ESR is a typical GGP area in China. Taking the ESR as a case study, the aims of this study are (1) to ascertain the changes in vegetation coverage and Land Use/Land Cover (LULC), and preliminarily evaluate the impact of land use changes on ecosystem function from 1990 to 2015; (2) to analyze the temporal–spatial characteristics of ecosystem health on the basis of the PSR model; and (3) to explain the impact of the GGP on ecosystem health on the basis of restored area data in order to develop better decisions on sustainability for the upper reaches of the Yangtze River basin.

## 2. Materials and Methods

### 2.1. Study Area

**Figure 1:**
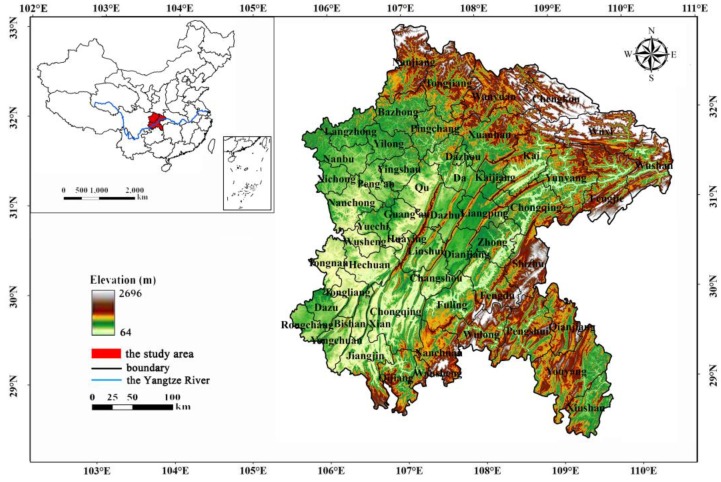
The location of the Eastern Sichuan Region (ESR).

The ESR, situated between E105°11′–110°11′ and N28°10′–32°45′, is located at the end of the upper reaches area of the Yangtze River in the eastern Sichuan Province in China with a total area of 130,143 km^2^ (Figure 1). The ESR contains five cities that govern 54 administrative counties. The ESR belongs to the mid-subtropical climate type, with an annual average temperature of 20 °C and precipitation of 1000 mm. The elevation ranges from 64 to 2696 m above sea level. The ESR is an important zone of water conservation and ecological shelter in the hilly area of the upper Yangtze River, leading it to a crucial targeted GGP implementation region.

### 2.2. Data Collection

**Figure 2:**
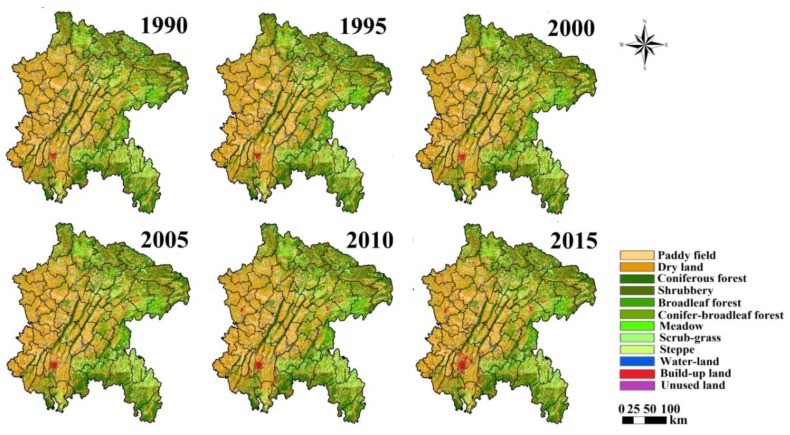
The land use types in this study area.

LULC data from the years 1990, 1995, 2000, 2005, 2010 and 2015 at a spatial resolution of 1 km was used as the basic data, which are obtained from the Data Center for Resources and Environmental Sciences, Chinese Academy of Sciences (RESDC). The land cover data were interpreted by visual interpretation using satellite images with an accuracy over 94.3% [29,30]. According to the national criteria announced by the Chinese government in 2007, LULC is divided into 6 primary types, including farmland, forestland, grassland, water land, build-up land and unused land. On this basis, we divided the vegetation coverage type in the study area according to the Vegetation Atlas of China (1:1,000,000). Farmland is divided into paddy field and dry land. Forestland is divided into coniferous forest, broadleaf forest, conifer–broadleaf forest and shrubbery. Grassland is divided into meadow, grassland and irrigation grass (Figure 2).

Annual normalized difference vegetation index (NDVI) data were the GIMMS (Global Inventory Modeling and Mapping Studies) NDVI 3 g in a bimonthly temporal resolution of 8 km spatial resolution from 1990 to 2015, which are available from the NASA Ames Ecological Forecasting Lab.

The annual average precipitation and temperature were obtained from the Data Center for Resources and Environmental Sciences, Chinese Academy of Sciences (RESDC). Per capita GDP of China comes from *The China Statistical Year Book*. The population data, the Engel coefficient data, the percentage of the urban population data, and the per capita GDP data come from *The Sichuan Statistical Year Book* and *The Chongqing Statistical Year Book*.

### 2.3. Methods

#### 2.3.1. Methods of Assessing LULC Changes

The basis of assessing the impact of the GGP on ecosystem health is to confirm that the GGP’s presence has changed the trend in vegetation coverage and to quantify the LULC changes. This implies a data analysis process that includes these main steps: (1) confirming the changes in vegetation coverage; (2) analyzing the LULC changes in detail, especially the land use types of farmland, forestland and grassland; and (3) preliminary assessing the impact of the LULC changes on ecosystem function.

The Mann–Kendall Trend Test Model can confirm the changes in vegetation coverage. This model determines the presence of abnormal factors influencing variables of NDVI by detecting potential turning points. It includes the following steps: (1) the annual NDVI data for the study region was calculated for 1990 to 2015 (for details, refer to the NDVI data processing in Section 2.3.3 in this study); and (2) the Mann–Kendall Trend Test Model used the annual NDVI data at 8 km spatial resolution from 1990 to 2015 on Matlab to judge whether there was an abrupt turning point. This technique calculated two statistical measures, which are the sequential values of NDVI. A forward sequential statistic is estimated using the original time series, and a backward sequential statistic is estimated in the same way but starting from the end of the series. The year of the intersection between the broken lines of the two statistics indicates a potential turning point, which is tested for its significance at the 95% level (*p* < 0.05). For details, refer to Chatterjee et al. [31,32]. If there is indeed a mutation point in sequence NDVI data, the trend in vegetation coverage suffered an unnatural disturbance. If tests identified the presence of a potential turning point, it is worth pursuing further analysis.

The land use transfer matrix model could further analyze the conversion of LULC, which helps to quantify and visualize the temporal–spatial changes in the vegetation coverage in detail. That can be done by spatial analysis tools of Arcgis 10.4 based on the land use data at 1 km spatial resolution.

The LULC change direction model (LCDM) relates to the preliminary assessment of the impact of LULC changes on ecosystem function [33]. The above-mentioned two test methods have been used to analyze the LULC changes in detail, but what do these LULC changes mean for the ecosystem? In this study, before conducting an ecosystem health analysis, it is necessary to make a preliminary assessment of the influence of these LULC changes on ecosystem function to predetermine whether the effect of the GGP on ecosystem health was good or bad, as well as to provide auxiliary proof of the validity of ecosystem health evaluation results. This is done by the LULC change direction model (LCDM), which can be defined as:

(1) $$LCDM=\frac{\sum_{i=1}^{n}\left[A_{ij}\times \left(D_{j}-D_{i}\right)\right]}{A}\times 100\%$$

where LCDM reflects the land use/land cover change value, i∈[1,n]; *i* reflects the *i*-th land use cover; *j* reflects *j*-th land use cover transformed from the *i*-th land cover in a specific period; $A_{ij}$ reflects the area of land use cover i transforming to land use cover j for the entire study region; $D_{i}$ represents the ecological level (Table 1) of the LULC type before the land cover change; $D_{j}$ represents the ecological level (Table 1) of the LULC type after the land cover change; and A reflects the total transformed area of all land use types for the entire study region in this period. When LCDM > 0, the impact of LULC change on ecosystem function is considered to be beneficial, and a higher LCDM value indicates better ecosystem functions. On the contrary, when LCDM < 0, the conversion is harmful, and the lower the LCDM value, the more negatively the ecosystem is functioning.

#### 2.3.2. The Method of Assessing Ecosystem Health Based on the PSR Model

The PSR model for ecosystem health evaluation was used at the county level for the years 1990, 1995, 2000, 2005, 2010 and 2015 in this study. We used the PSR model proposed by Liao [18] to construct an ecosystem health index (EHI) evaluation system based on the land use data and population. The PSR model has three dimensions (pressure, state, and response) and seven indicators (population density (PD), the landscape fragmentation index (LFI), the normalized difference vegetation index (NDVI), the landscape diversity index (LDI), the average patch area index (APAI), the ecosystem service value (ESV), and the ecological resilience (ER)) (Table 2). Among three dimensions, “response” refers to the regression analysis between the restored areas, reflecting the response of human, and the increased EHI values. GGP implementation started from 1999 in the ESR. Because the time scale of “response” is not as the same time scale of “pressure” and “state”, regression analysis of the “response” started from 2000.

**Figure 3:**
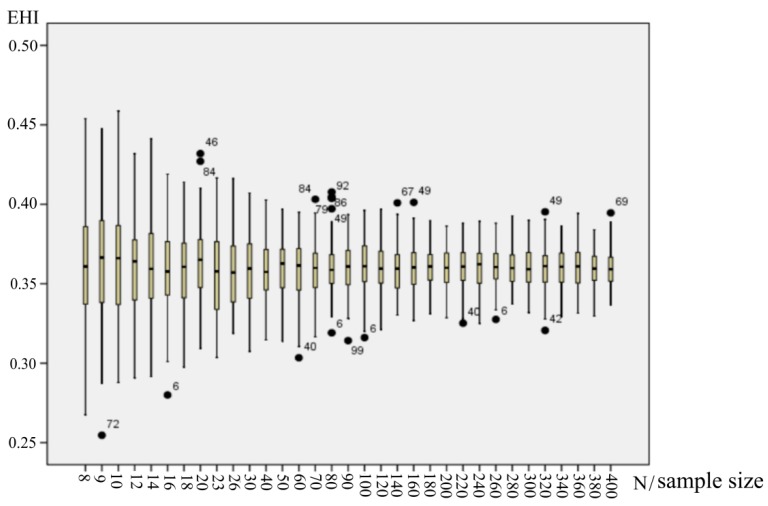
The box-plots of the ecosystem health index (EHI) based on Monte Carlo simulation. (1) Each box-plot represents the dispersion of 100 EHI values obtained in 100 times simulation of a specific sample size (N); (2) the black dots represent the outliers; the number next to the black dots represents the ordinals of 100 times simulation.

The Analytic Hierarchy Process (AHP) method and Monte Carlo method were used to identify the weight of the index. Fifteen ecological experts with rich experience in ecology were invited to complete a questionnaire survey to quantify the factors influencing ecosystem health by providing weights to each index based on the AHP calculation method. The questionnaire results of the eleven experts passed the consistency test, and we gained eleven primary group weights [34]. Following this, a Monte Carlo simulation conducted in Matlab was used to eliminate the uncertainties which may have been introduced by the experts’ experience. The data stimulation process mainly included the following steps: (1) 10,000 group weight samples were generated based on the mean and standard deviation of each indicator using Matlab; (2) we randomly determined the weight sample size as N (N = 8, 9, 10, …, 400), and then took N group weight samples at one time to randomly sample 100 times from those 10,000 group weight samples. Each index weight was averaged based on these N (N = 8, 9, 10, …, 400) samples; (3) 100 times weights were respectively adapted to a county in the study area to calculate the EHI; (4) a box-plot of the EHI was drawn as shown in Figure 3; Figure 3 shows that the simulation results tended to be stable with a sample size of 50. A sample capacity of 200 was selected to calculate the final weight of each index in this study (Table 2).

The combination of all the indices is taken as the EHI. In the multi-index evaluation system, due to the different attributes of each evaluation index, such as different dimensionality and orders of magnitude, we ensured the reliability of the results by standardizing the raw indicator data. The formula is as follows:

(2) $$HI=\overset{n}{\underset{i=1}{\sum }}w_{i}x_{i}$$

where i represents the number of indicators, $x_{i}$ means the value of a specific indicator, and $w_{i}$ is the weight of the *i*-th indicator. A greater index value always implies better ecosystem health.

#### 2.3.3. Indicators of the PSR Framework

Landscape fragmentation, the degree of the fragmentation of the landscape, reflects the complexity of the spatial structure of the landscape. The formula is as follows:

(3) $$LFI=\left(N_{t}-1\right)/N_{c}$$

where LFI reflects the landscape fragmentation of the study region; $N_{t}$ reflects the total patch numbers; and $N_{c}$ reflects the ratio between the minimum patch area and the total area of the study region.

The NDVI reflects the vegetation coverage. The data calculation process used in this study included three main steps [35]: (1) in order to remove the interference from clouds, atmosphere, and the solar elevation angle, the NDVI of the fortnightly data set was preprocessed by Max Value Composites (MVCs) to obtain the monthly NDVI value; (2) we averaged the value of the growing season from April to August as the annual NDVI value to eliminate the effects of extreme months; (3) NDVI was averaged on the full area. Equations are as following:

(4) $$NDVI_{ij}=max\left(NDVI_{ij1},\;NDVI_{ij2}\right)$$

(5) $$NDVI_{i}\frac{\sum_{j=1}^{n}NDVI_{ij}}{5},\;j\in \left[4,8\right]$$

(6) $$NDVI=\overset{n}{\underset{j=1}{\sum }}NDVI_{j}$$

where i stands for the *i*-th pixel; j stands for the *j*-the month; *j*1 and *j*2 stands for the first and second half of the month, respectively. $NDVI_{ij}$ represents NDVI value of the *i*-th pixel for the *j*-th month; $NDVI_{ij1}$ and $NDVI_{ij2}$ is the NDVI value of the *i*-th pixel for the first and the second half of the *j*-th month, respectively. $NDVI_{i}$ stands for the NDVI value of the *i*-th pixel for the specific year. NDVI stands for the NDVI value of the county under study for the specific year. The above steps are based on the spatial analysis tool of Arcgis 10.4 (Environment System Research Institute, Redlands, CA, USA).

The complexity of the ecosystem structure is measured by the landscape diversity (Shannon’s diversity index) and the average patch area using the tool of Fragstats 4.2 (Oregon State University, Corvallis, OR, USA). The average patch area is expressed as Equation (7):

(7) $$APAI=\frac{A}{N}$$

where APAI reflects the average patch area index, A represents the total area of patch in the landscape of the study area, N represents the total number of patch in the study area.

The method used to calculate the ecosystem service value proposed by Xie [36] at national level is not suitable for calculating the value at regional level. So, we amended the ecosystem service value model depending on the net primary productivity of natural vegetation to be more suitable for the study area, in accordance with Li [37]. The equations are:

(8) $$ESV=\sum \left\{A_{k}\times VC_{k}\times \frac{NPP_{s}}{NPP_{cn}}\times \left[2/\left(1+e^{2.5-\frac{1}{En}}\right)\times \left(GDP_{ms}/GDP_{m}\right)\right]\right\}$$

(9) $$NPP=3000\left\{1-e^{-0.0009695\left(V-20\right)}\right\}$$

(10) $$v=\frac{1.05Pre}{\sqrt{1+{\left(1+1.05Pre/L\right)}^{2}}}$$

(11) $$L=3000+25Tmp+0.05Tmp^{3}$$

(12) $$En=En_{r}\times \left(1-P_{u}\right)+En_{u}\times P_{u}$$

where ESV is the ecosystem services value; $A_{k}$ implies the area of k-th type of land use; $VC_{k}$ is the ecosystem services value per unit area of the k-th type of land use in China (yuan/ha) [36]; $NPP_{s}$ represents the net primary productivity of natural vegetation in the study area, and $NPP_{cn}$ represents the net primary productivity of natural vegetation in China. NPP implies the net primary production potential of natural vegetation (t/ha/a); v is the annual actual evapotranspiration (mm); Pre is the annual precipitation (mm); L represents the annual mean evapotranspiration (mm); Tmp represents the annual average temperature (°C); En is the Engel coefficient of the study area in 2002; $En_{r}$ and $En_{u}$ are the Engel coefficient of rural and urban areas, respectively in 2002; $P_{u}$ represents the percentage of the urban population in 2002; ${GDP}_{ms}$ is the per capita GDP of the study area in 2002 (yuan/person); and $GDP_{m}$ is the per capita GDP of China in 2002 (yuan/person).

Ecological resilience refers to the ability of the ecosystem to return to a stable state after an external disturbance has disappeared. This paper uses the ER index to quantify the ability of the ecosystem to return to normal state after disturbance using the following equation:

(13) $$ER=D\overset{n}{\underset{i=1}{\sum }}S_{i}\times E_{i}$$

where D represents the landscape diversity index; $S_{i}$ is the area ratio of each landscape type; $E_{i}$ is the resilience value of the landscape type *i*, in accordance with Du et al. [28].

#### 2.3.4. Assessing the Impact of the GGP on the EHI

**Figure 4:**
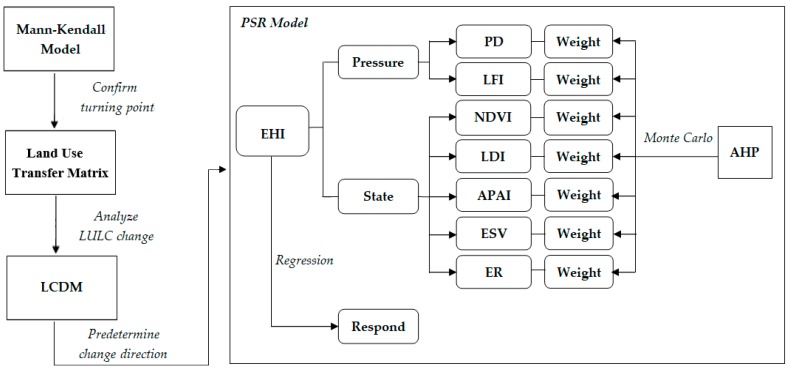
Systematic overview of the methods.

In this study, the response part of the PSR model was driven by the GGP. The EHI is expressed as the combination of pressure and state indicator. In order to assess the impact of the GGP on the EHI, we use regression analysis between the restored forest areas (Δ Areas) and the increased EHI values (Δ EHI) at the county level using the tool in SPSS 19 (IBM, Armonk, NY, USA). The time scale of regression analysis is in three periods of 2000 to 2005, 2005 to 2010, and 2010 to 2015. All the methods used in this study refer to Figure 4.

## 3. Results

### 3.1. The Changes in LULC at Different Periods

#### 3.1.1. Transfer Matrix

**Figure 5:**
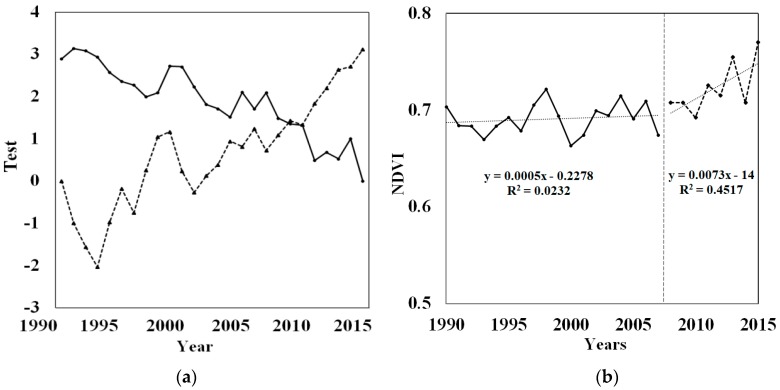
The changes in annual normalized difference vegetation index (NDVI) from 1990 to 2015: (**a**) Abrupt changes in the annual NDVI from 1990 to 2015 derived from the Mann–Kendall Trend Test Model test statistics, where the year of the intersection is the potential turning point; (**b**) Variation in the annual NDVI and linear trends for the two periods.

The Mann–Kendall Trend Test Model was proposed to verify whether a turning point of vegetation coverage change exists. The results are shown in Figure 5. A year with an intersection between the broken lines of the forward and backward sequential NDVI statistics was indeed identified (*p* < 0.05) (Figure 5a), and the change in NDVI showed an increasing trend (Figure 5b). This test confirmed the presence of the turning point, which concluded that unnatural factors certainly had an impact on improving the vegetation coverage.

**Figure 6:**
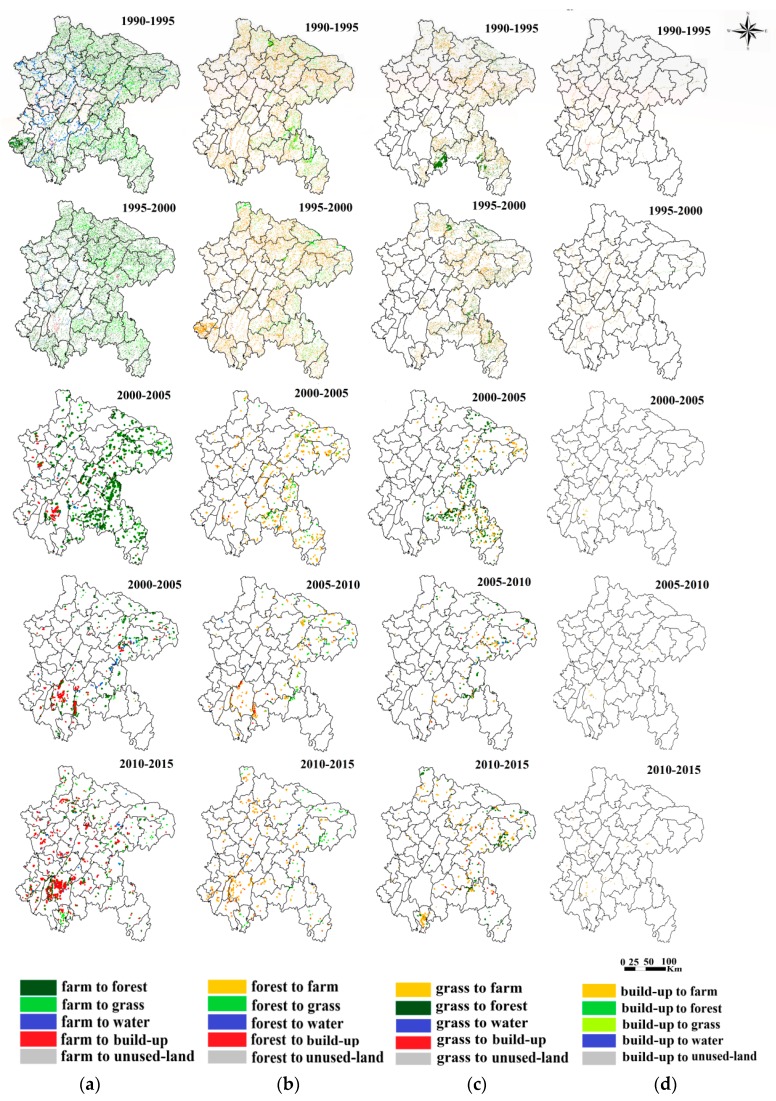
Transfer matrix map of land use: (**a**) Farmland; (**b**) Forestland; (**c**) Grassland; (**d**) Water land.

To further analyze the temporal–spatial conversion of the vegetation coverage, the transfer matrix map of LULC was created for the study area at a 1 km spatial resolution (Figure 6). Significant changes were observed in 2000, when there was a turning point.

From 1990 to 2000, the transfer dynamic was more active than changes in the following period (Figure 6). Additionally, in the period from 1995 to 2000, the area of land use changed significantly. The farmland areas increased by 969 km^2^. The areas of forestland, grassland and water land decreased by 473, 579, and 55 km^2^, respectively.

Since 2000, the transfer dynamic has dropped greatly (Figure 6). Meanwhile, the area of farmland decreased significantly but forest increased significantly. From 2000 to 2005, the transfer dynamic was mainly gathered to the east of the study area, leading to a decrease in farmland area, while the forest and water land areas increased. Though grassland areas shrank consistently, the degradation rate of grassland dropped apparently since 2000. In addition, the transfer dynamic of water land near the Yangtze River was controlled effectively. From 2005 to 2015, the build-up of agglomeration spread outwards from Chongqing quickly because of urbanization.

#### 3.1.2. The LULC Change Direction

The analysis of the transfer matrix map showed that the farmland, forestland, and grassland areas have obviously changed. Since 2000, the forestland area has increased significantly. This trend is definitely affecting the changes in ecosystem function. Therefore, it is necessary to make a preliminary assessment of the influence of LULC changes on ecosystem health based on the changes in the LULC model (LCDM).

We calculated the LCDM values based on Equation (1), and the results were as follows: LCDM_1990–1995_ = 0.56%, LCDM_1995–2000_ = 0.14%, LCDM_2000–2005_ = 8.48%, LCDM_2005–2010_ = 7.26%, LCDM_2010–2015_ = 3.06%. The results show that the LCDM value increased significantly after 2000, implying changes in LULC had a beneficial impact on ecosystem function in the study area since 2000. This also preliminarily confirms that the GGP has definitely been advantageous for ecosystem health in this study area.

### 3.2. Evaluating Ecosystem Health Based on the PSR Framework

#### 3.2.1. Changes in the Pressure and State Indicators

Overall, in this study region, indicators reflecting the pressure (PD and LFI) decreased (a higher pressure) from 1990 to 2015, while the indicators of state (PD, LFI, NDVI, LDI, APAI, ESV and ER) decreased from 1990 to 2000 but increased from 2000 to 2015 (Table 3).

**Figure 7:**
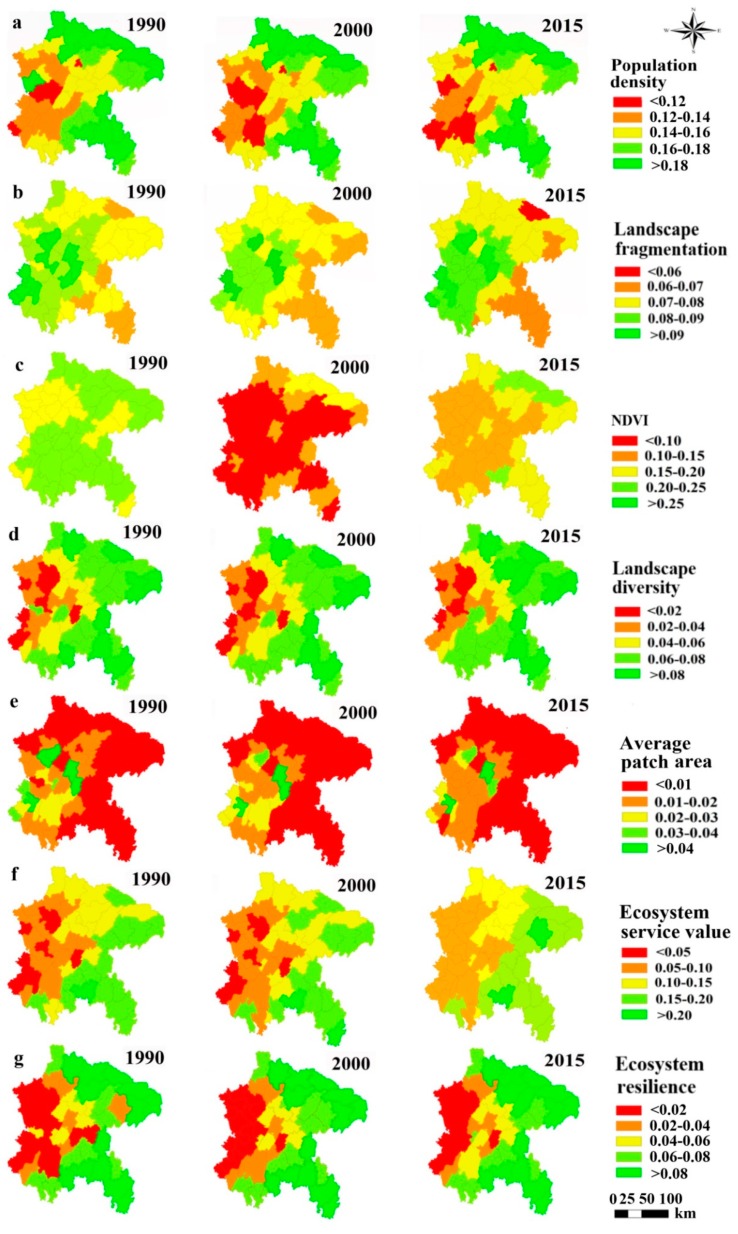
The spatial distribution of different indices at the county level based on standardized data for the years 1990, 2000 and 2015: (**a**) PD; (**b**) LFI; (**c**) NDVI; (**d**) LDI; (**e**) APAI; (**f**) ESV; (**g**) ER.

The pressure is considered to indicate the more intensive pressure imposed by humans and the greater degradation of ecosystem functions to show as negative values. Firstly, the proportion of counties where PD and LFI decreased were 94 and 67% respectively from 1990 to 2015 (Figure 7), indicating a high level of pressure induced by humans throughout the study period. Second, the PD pattern had a low value in the west but a high value in the east, while the LFI was completely opposite in terms of its spatial pattern, because the population and farmland was mainly concentrated in the west, resulting farmland in the west were more connected than that in the east.

The state indicators were considered to be more beneficial for ecosystem functions conserved at higher values. The results of most state indicators showed a positive trend except APAI from 2000 to 2015 (Table 3 and Figure 7). The proportion of counties with a declining NDVI reached 100% from 1990 to 2000, while only Nanchuan showed a decrease in the next state (2000–2015). For LDI, 33% of counties changed from negative to positive before and after 2000. The ESV value was raised in 28% of counties and dropped in only two counties from 2000 to 2015. The ratio of counties with decreasing ER was 41% before 2000. But the ER in 96% counties increased after 2000. Compared to the above indices, the condition of APAI was not good, showing a constant decrease (Table 3). From the perspective of spatial distribution, APAI showed a spatial pattern with high value in the west and low values in the east, similar to the pattern of LFI (Figure 7e), which was reversed completely for the other state indicators. This implies that the ecosystem condition is good but connectivity is poor in the east.

#### 3.2.2. Changes in the EHI

**Figure 8:**
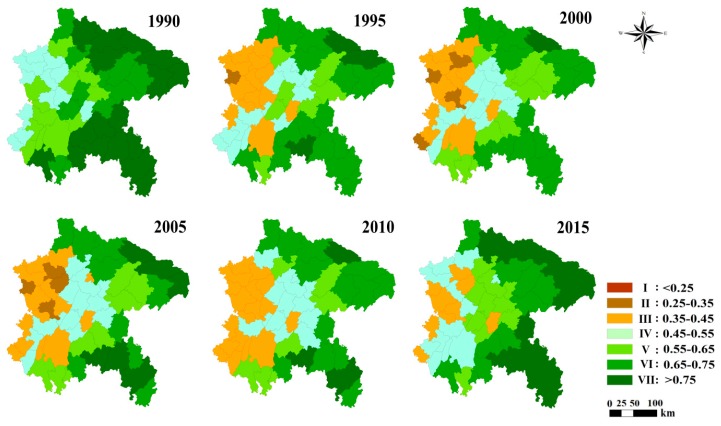
Assessment of the EHI for the study area from 1990 to 2015.

The results of seven ecological health indices based on pressure and state indicators for the study period are depicted in Figure 8.

At the whole region scale, the EHI consistently changed to negative from 1990 to 2000 but turned to positive significantly in the following phase (2000–2015) (Table 3). At the county scale, the proportion of counties with high health degree grades (V, VI and VIII) decreased from 1990 to 2000, but increased from 2000 to 2015. In contrast, the proportion of counties with low health degree grades (I, II, III) increased from 1990 to 2000, and decreased from 2000 to 2015. To sum up, ecosystem health in the study area was improved.

In terms of spatial variation (Figure 8), the following characteristics were identified: (1) counties with a high EHI value were mainly distributed in the eastern part of the ESR, and counties with a low EHI value were mainly gathered in the northern and western parts. (2) From 1990 to 2000, the number of counties with high EHI values decreased, and the number of counties with low values increased. The situation reversed completely in the following period (2000–2015) and the EHI values of most counties continued to be higher than in the former phase. (3) The intensity of ecological recovery carried out by the government was stronger in more devastated areas. This can be confired by the phenomenon that the county with the fastest decline in the EHI in 1990–2000 and the fastest increase in the EHI in 2000–2015 was Fulin. (4) Forest is vital to the ecosystem’s overall condition. The county with the highest EHI value was always Chengkou from 1990 to 2010, but this was replaced by Wulong in 2015. The county with the lowest EHI throughout the study period was Xichong. This implies the ecosystem conditions of Chengkou and Wulong were better than the conditions of Xichong. This is mainly because the proportions of forest areas in Wulong and Chengkou are much higher.

### 3.3. Impact of the GGP on the EHI

**Figure 9:**
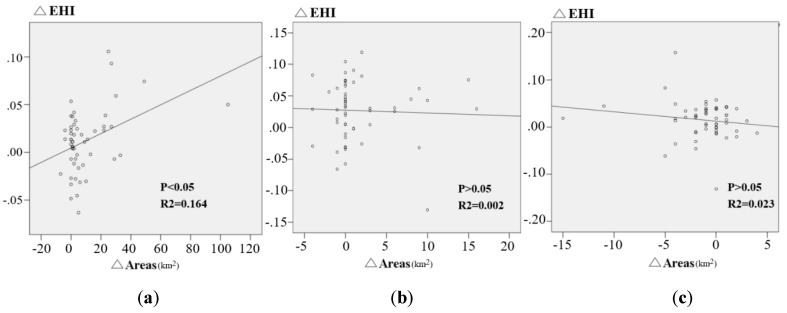
Relationship between the EHI and ecological restoration areas from 2000 to 2015 at the county level, ∆EHI represents changes in the EHI: (**a**) 2000–2005; (**b**) 2005–2010; (**c**) 2010–2015.

From 2000 to 2005, the initial period of GGP implementation, the correlation between increased EHI and the restored areas was significant (*p* < 0.05, R^2^ = 0.164), but no significant correlation was shown in the following phase (*p* > 0.05) (2005–2010, 2010–2015) (Figure 9). This indicates that the GGP apparently improved ecosystem health in the ESR from 2000 to 2005; however, this correlation disappeared in the following period, suggesting that health was improved through not only afforestation but also other means at the same time.

## 4. Discussions

### 4.1. The GGP Changed the Transfer Direction of LULC

In this study, the trend in NDVI in the ESR was altered by abnormal factors (Figure 5), which may largely be attributed to the GGP. First, according to Liu’s research [19], the Mann–Kendall Trend Test Model testing statistics can determine whether there are mutations in the sequence data. The turning point identified by this method showed that NDVI suffered unnatural mutations (Figure 5a) during the study period. Second, the GGP has been actively implemented in the study area since 1999. The major impact of the GGP has been an increase in vegetation coverage, which is supported by previous results [17,38,39]. This is constant with the results of Mann–Kendall Trend Test Model, which showed that the vegetation coverage rate rose significantly (Figure 5b). Further, the climatic conditions have basically been stable in recent decades. Additionally, the main changes in vegetation coverage in this research were an increase in forestland and a decrease in farmland. Using a transfer matrix map, we identified the change in NDVI was mainly caused by the GGP.

The purpose of the GGP is to covert farmland to forestland and grassland [5,6,7]. In this study, the GGP was shown to affect the transfer direction of LULC (Figure 6), especially forestland, farmland, grassland and water land. According to the transfer matrix map, we bring the following new insights into the traits of the GGP.

From the perspective of temporal variety, a certain staged characteristic exists in the process of the GGP and different tasks are conducted at different stages. First, the program has effectively managed the transfer dynamic of land use. Before the GGP, the transfer dynamic was more active than in following the phase (2000–2015), largely as a result of massive deforestation and reclamation. Fortunately, this situation then disappeared. Second, as expected, the GGP was associated with significant increases in forestland and decrease in farmland as shown in Figure 6. Additionally, the government controlled the degradation of grassland to slow down the rate of grassland area reduction in the period of GGP implementation. We can see that the increase in forest area was faster in the initial phase (2000–2005), and slowed down in the following period (2005–2015). The years after 2011 represent the later consolidation stage of the program, during which the main tasks were woodland protection and management and local reforestation rather than purely afforestation [40]. Third, the GGP had an additional effect of increasing water land area. It effectively controlled the decreasing water land area. Previous studies also showed that it had a potential impact on water resource [41]. Large-scale afforestation may not only promote the digging up of streams to improve the water environment, but also plays an important role in water conservation. The above phenomena are all consistent with the implementation stage of the project.

From the perspective of spatial distribution, the GGP has territoriality features. Figure 5 shows the intensity of the project was the highest from 2000 to 2005, and the key implementation region of afforestation was in the east area of the ESR (Figure 6). This can be interpreted as being due to forests preferring to grow in the eastern region with its rugged terrain. Additionally, the western part of the research area has relatively flat terrain, where large farmlands exit as China’s grain production base. When carrying out the GGP, more attention should also be paid to controlling the red line area of farmland.

Finally, on the basis of the LCDM results, the variety in the transfer matrix under the influence of the GGP was positively related to ecosystem function. In general, the GGP has effectively changed the usage of land and is beneficial to ecosystem function.

### 4.2. The GGP Enhanced the EHI of the ESR

An ecological health assessment is a process that estimates the comprehensive ecosystem conditions related to human pressure and the state of the ecosystem, as well as the human response to ecosystems degradation. Despite this, the variation in LULC under the influence of the GGP had a positive effect on ecosystem function. Given the LCDM results, the EHI results characterizing the pressure and state caused by the GGP were proved. A previous study showed that ecological restoration enhances the ecosystem health of ecosystems [18], and their results were similar to those in this study.

From a temporal perspective of the EHI pattern, this study showed a clear constant negative trend in the EHI from 1990 to 2000, but this changed to a positive direction in the following phase (2000–2016) (Table 3, Figure 8). That was mainly attributed to the GGP, which has led to a great increase in forestland and a decrease in farmland. The indices (LDI, ESV and ER) dominating at the state level had low values for farmlands but high values for forests, ultimately explaining the general increase in the EHI from 2000 to 2015. However, the pressure indices (PD and LFI) decreased to some degree (high pressure), but their negative impact was offset by the positive development of the remaining indices. These are the major reasons that explain the EHI towards a higher value after the GGP.

In terms of the spatial pattern, the close relationship between the population and the land use is associated with the EHI spatial pattern. The western and eastern terrains differ significantly in the ESR. The west is plain, and the east is mountainous (Figure 1). Different geographic environments lead to the density differences of population and road network in between eastern and western regions. Acute topographic undulation is not good for economic development and human habitation. Therefore, the eastern region with acute topographic undulation is associated with a large area of forestland and grassland leading to a sparse population, blocked transportation and a low level of urban development. The EHI value of the eastern region was found to be higher (Figure 8). The western plateau area, by contrast, is associated with accelerated economic development and the urban expansion. The economic growth promotes the increase in population and the construction of roads. The functions of ecosystem services would be in serious recession, leading to ecosystem health degradation. The EHI value of the eastern region was lower than for the western region (Figure 8).

Regarding the regression analysis, the changes in the EHI were clearly positively associated with the restored forestland areas (*p* < 0.05, R^2^ = 0.164) in the early stage of the GGP (2000–2005), implying that the GGP has certainly significantly enhanced the EHI in the ESR. In the following phase (2005–2015), changes in the EHI were not significantly affected by the restored forestland areas. Certainly, in this period (2005–2015), the EHI showed a positive change trend throughout the whole area (Table 3 and Figure 8), but the increased rate of restored forestland areas slowed down a lot compared with the former stage (2000–2005) (Figure 6). This may, in part, be explained by the greater focus on promoting the growth of woods than afforestation purely to improve the ecological conditions in the later stage of the GGP (2005–2015) [40], which shows that the staged trait effectively improves environmental conditions in different ways at different stages of the GGP. In summary, the different measures taken at different stages of the GGP all effectively enhanced the EHI in the ESR.

### 4.3. Other Factors Influencing the EHI and Sustainability Decisions

As shown in this research, the EHI showed a clear pattern of increasing from west to east in the ESR (Figure 8). The EHI is also affected by many other factors which are not accounted for in the indictors of the EHI system. These can be summarized as follows:

(1) Public pressures, which are closely associated with land use pattern, establish the primary spatial pattern of the EHI. The flat topography is suitable for agricultural development and rapid urban expansion. Flat topography has led to this study area being occupied by large farmland and built-up land (Figure 2) as a result a lower ecosystem service values in western areas leading to a lower EHI (Figure 8). Inversely, the EHI value in the eastern area is generally higher.
(2) The temporal pattern of the EHI is associated with government response. In this study area, the EHI values were apparently improved by government response throughout the study period (Figure 8 and Figure 9). The average negative impact of public pressure was generally offset by the positive development of government response. Therefore, if public pressure is high and the government response does not work properly, the EHI value would decrease—otherwise, the EHI value would increase throughout the study period. As expected, governments usually enhance the intensity of ecological recovery in more devastated areas with high public pressure—as a result, the EHI value could be generally improved in most counties of the study area (Figure 8).
(3) Technological advancement can also influence the effectiveness of the GGP to some degree. GGP implementation is usually accompanied by the technical means, such as concreting bare land or covering land with jute nets to avoid splash erosion and gulley formation. Pervious research has showed that the GGP could enhance soil fertility and control soil erosion [5,15]. However, this study does not involve the effectiveness evaluation on soil conservation, so we will pay attention to this in our future work.

Therefore, for governments, the adoption of reasonable eco-environmental restoration and resource management strategies by the government plays an important role in sustainable development. Here are some suggestions:

(1) Flat topography is usually suitable for agricultural development and rapid urban expansion. Additionally, the intensity of ecological recovery is particularly enhanced on slopes and in areas with worse ecosystem conditions. This shows that policy makers should pay attention to not only the rational planning of urban and farmland regions but also increase the protection of ecological land, especially in the eastern part of the ESR.
(2) Research results show that APAI and LFI were low, especially in the east region of the ESR, indicating poor forest connectivity. Therefore, the government should improve the connectivity of artificial forest and grassland to increase the EHI.
(3) Unhealthy areas of the ESR generally show low vegetation coverage, which is mainly caused by human activities including the reclamation of land and the construction of urban land. Therefore, a reasonable recommendation is to restore the grassland ecosystem in the southeast grassland region, afforest the arbor and shrub forests in the northeast region, and form farmland ecosystems and urban systems under the protection of the forest network in the process of carrying out the GGP to attain sustainable development goals.

## 5. Conclusions

The Eastern Sichuan Region (ESR), one of the key pilot regions of GGP implementation in China, has significant ecological protection as an ecological shelter zone of the upper reaches of the Yangtze River basin. Assessing the effects of the GGP against the background of human pressure and ecosystem state factors is vital to allow the government to form reasonable eco-environmental restoration and resource management strategies. In this article, the Mann–Kendall Trend Test Model was used to ascertain the changes in vegetation coverage. The transfer matrix was used to explore the changes in land use/land cover (LULC). The LULC change direction model (LCDM) was used to preliminarily assess the impact of LULC changes on the ecosystem. The Pressure–State–Response model (PSR), reflecting human pressure and the ecosystem state, was applied to analyze the spatial–temporal characteristics of the ecosystem health index (EHI). The results showed that with GGP implementation in the study area, vegetation coverage changed significantly (*p* < 0.05). The main variation in LULC was an increase in forestland and water land and decrease in farmland. Additionally, the rate of grassland degradation decreased. The variation in LULC moved ecosystem function in a positive direction. With the backgrounds of human pressure and the state of the ecosystem, the spatial distribution of the EHI is influenced by the pattern of land use. The eastern region is associated with a large area of forestland and grassland had a low population density and a low degree of land use exploitation, resulting in a high EHI value. The situation was completely opposite in the western region. Regarding temporal changes, in spite of a decreasing pressure indicator, we found large-scale increases in the EHI, which were dominated by the GGP. Additionally, there was a clear correlation between the increased EHI values and the increased forest areas of the GGP in the former stage (2000–2005), but this correlation disappeared in the last stage (2005–2015). This pattern indicates certain stage and territoriality characteristics exist in the process of the GGP. The ultimate conclusion that can be drawn from our study is that the GGP has caused a large-scale transformation of farmland into forestland, grassland and water land, which has led to a general increase in the EHI.

## Figures and Tables

**Table 1:** Ecological level of the different land use [33].

**LULC**	**Farmland**	**Forestland**	**Shrub-Land**	**High-Density Grassland**
Ecological levels	0.11	0.245	0.147	0.125
**LULC**	**Mid-Density Grassland**	**Low-Density Grassland**	**Water Land**	**Unused Land**
Ecological levels	0.063	0.018	0.282	0.01

**Table 2:** The Pressure-State-Response (PSR) framework and the weight of each indictor.

Target Level	Standard Level	Weight	Indicator Level	Weight
Ecosystem health index (EHI)	Pressure	0.3	Population density (PD)	0.20
			Landscape fragmentation index (LFI)	0.10
	State	0.7	Normalized difference vegetation index (NDVI)	0.24
			Landscape diversity index (LDI)	0.09
			Average patch area index (APAI)	0.05
			Ecosystem service value (ESV)	0.22
			Ecological resilience (ER)	0.10
	Response	---	Restored areas	----

**Table 3:** Changes in different indices in the Pressure–State–Response (PSR) framework from 1990 to 2015 in the ESR at the region scale.

Indicator Level	Index Level	Year					
		1990	1995	2000	2005	2010	2015
Pressure	PD	0.1568	0.1554	0.1540	0.1526	0.1547	0.1539
	LFI	0.0833	0.0839	0.0827	0.0825	0.0813	0.0813
	Total	0.2401	0.2393	0.2367	0.2351	0.2360	0.2352
State	NDVI	0.0387	0.0280	0.0000	0.0266	0.0280	0.1028
	LDI	0.0750	0.0755	0.0753	0.0758	0.0769	0.0788
	APAI	0.0116	0.0118	0.0111	0.0110	0.0106	0.0101
	ESV	0.1252	0.1293	0.1257	0.1274	0.1289	0.1289
	ER	0.0696	0.0704	0.0700	0.0706	0.0715	0.0730
	Total	0.3201	0.3150	0.2821	0.3114	0.3160	0.3937
EHI	-	0.5602	0.5544	0.5189	0.5465	0.5521	0.6289
